# PyCompound: An Open-Source Python Package for Spectral-Library Matching in Mass Spectrometry-Based Metabolomics

**DOI:** 10.3390/metabo16080585

**Published:** 2026-08-18

**Authors:** Hunter Dlugas, Xiang Zhang, Xun Bao, Jing Li, Ikuko Kato, Seongho Kim

**Affiliations:** 1 Biostatistics and Bioinformatics Core, Karmanos Cancer Institute, Wayne State University, Detroit, MI 48201, USA; 2 Department of Chemistry, University of Louisville, Louisville, KY 40292, USA; 3 Department of Oncology, School of Medicine, Wayne State University, Detroit, MI 48201, USA; 4 Pharmacology and Metabolomics Core, Karmanos Cancer Institute, Wayne State University, Detroit, MI 48201, USA; 5 Populations Sciences in the Pacific Program, Epidemiology Division, University of Hawaii Cancer Center, Honolulu, HI 96813, USA

**Keywords:** chemoinformatics, compound annotation, entropy, mass spectrometry, metabolomics, spectral library matching, similarity measures

## Abstract

Spectral-library matching is a widely used approach for compound annotation in mass spectrometry (MS)-based metabolomics, yet annotation performance is influenced by spectrum preprocessing, parameter selection, and similarity-measure choice. We present PyCompound, an open-source Python package for spectral-library matching with flexible preprocessing, parameter optimization, and diverse similarity measures for both nominal-resolution and high-resolution mass spectrometry data. PyCompound implements six preprocessing procedures, nineteen similarity measures, including the newly developed Rényi entropy similarity in this work for spectral-library matching, user-defined composite similarity scores, and automated parameter optimization using grid search and differential evolution. The software supports common spectral formats and is accessible through a Python API, command-line interface, and interactive Shiny application. The software was validated using public GNPS LC-MS/MS and WebNIST GC-MS datasets, where the newly developed Rényi entropy similarity demonstrated annotation performance comparable to that of the established Shannon and Tsallis entropy similarity measures. PyCompound provides a flexible platform for practical compound annotation through configurable preprocessing workflows, diverse similarity measures, and automated parameter optimization.

## 1. Introduction

Mass spectrometry (MS) is a leading analytical platform for metabolomics, enabling comprehensive characterization of small molecules in biological and environmental samples. Despite substantial advances in MS instrumentation, compound annotation, often referred to as compound identification, remains a major bottleneck because many detected features cannot be confidently assigned to known metabolites. Consequently, considerable effort has been devoted to developing computational methods that improve the accuracy, robustness, and reproducibility of compound annotation workflows [1,2].

Among available annotation strategies, spectral-library matching remains one of the most routinely used and experimentally grounded approaches [3,4]. In this approach, a query spectrum is compared with reference spectra from experimentally characterized compounds, and candidate annotations are generated according to a selected similarity measure. Spectral-library matching is commonly employed in gas chromatography–mass spectrometry (GC-MS) and liquid chromatography–tandem mass spectrometry (LC-MS/MS) workflows and utilizes major public spectral resources, including GNPS, MassBank, MoNA, HMDB, METLIN, and NIST libraries [3,5].

Although conceptually straightforward, spectral-library matching depends on multiple factors, including reference-library coverage, acquisition conditions, preprocessing procedures, parameter settings, and the similarity measure to compare spectra [3,6,7]. Consequently, annotation accuracy can vary substantially across datasets even when the same similarity measure is applied [5,8]. Recent studies have further shown that preprocessing choices and acquisition parameters can influence annotation performance as much as, or more than, the similarity measure itself [6]. Collectively, these findings highlight the need for software that provides flexible preprocessing workflows, diverse similarity measures, and parameter optimization rather than relying on fixed processing pipelines.

The distinction between nominal-resolution and high-resolution MS adds further complexity to compound annotation. GC-MS datasets acquired using electron ionization typically exhibit highly reproducible fragmentation patterns and benefit from extensive reference libraries. In contrast, LC-MS/MS datasets acquired using electrospray ionization often display greater variability arising from differences in instrument settings, collision energy, precursor selection, adduct formation, and library composition. Consequently, LC-MS/MS workflows generally require more flexible preprocessing and parameter optimization.

Cosine similarity has historically been the dominant similarity measure for spectral-library matching because of its computational efficiency and strong empirical performance [4,9]. More recently, entropy-based similarity measures have provided alternative information-theoretic approaches for comparing normalized spectra and have demonstrated competitive performance for small-molecule spectral matching [3,6,7]. Accordingly, no single similarity measure is expected to be universally optimal across all datasets and analytical conditions.

Several software tools support spectral processing and compound annotation in metabolomics, including Matchms, MetaboAnnotation, metID, and ShinyMetID [10,11,12,13]. These packages provide valuable functionality for routine metabolomics workflows but, as summarized by the selected functional characteristics in Table S1, generally place less emphasis on combining flexible preprocessing, parameter optimization, diverse similarity-analysis options, and user control over spectral-library matching within a single framework. The comparison is intended to characterize differences in scope and functionality rather than to provide a comprehensive assessment or overall ranking of these tools.

Recent advances in machine learning have introduced new opportunities for compound annotation through candidate structure prediction and generation of large in silico spectral libraries [14,15,16,17]. Machine-learning-based spectral-similarity methods, including Spec2Vec [7] and MS2DeepScore [18], use learned spectral representations and may capture relationships that are not explicitly represented by conventional similarity measures. PyCompound is intended to complement these approaches by providing transparent and configurable preprocessing, similarity calculations, composite scores, and parameter optimization. Spectral-library matching nonetheless remains essential in metabolomics workflows because similarity-based ranking is widely used to prioritize or validate candidate annotations derived from experimental or computational spectra [7,19].

To address these challenges, we present PyCompound, an open-source Python package for spectral-library matching in MS-based metabolomics. The software implements six preprocessing procedures, nineteen similarity measures (four continuous and fifteen binary measures), including the newly developed Rényi entropy similarity, user-defined composite similarity scores, and automated parameter optimization within a unified framework supporting both nominal-resolution and high-resolution MS data. Designed for practical compound annotation, PyCompound enables users to tailor preprocessing workflows, optimize analysis parameters, and select similarity measures appropriate for their experimental data. To demonstrate its practical utility, the software was applied to public GNPS LC-MS/MS and WebNIST GC-MS benchmark datasets, and the annotation performance of the newly developed Rényi entropy similarity was evaluated relative to established similarity measures.

## 2. Characteristics and Implementation

### 2.1. Software Design and Functionality

**Figure 1:**
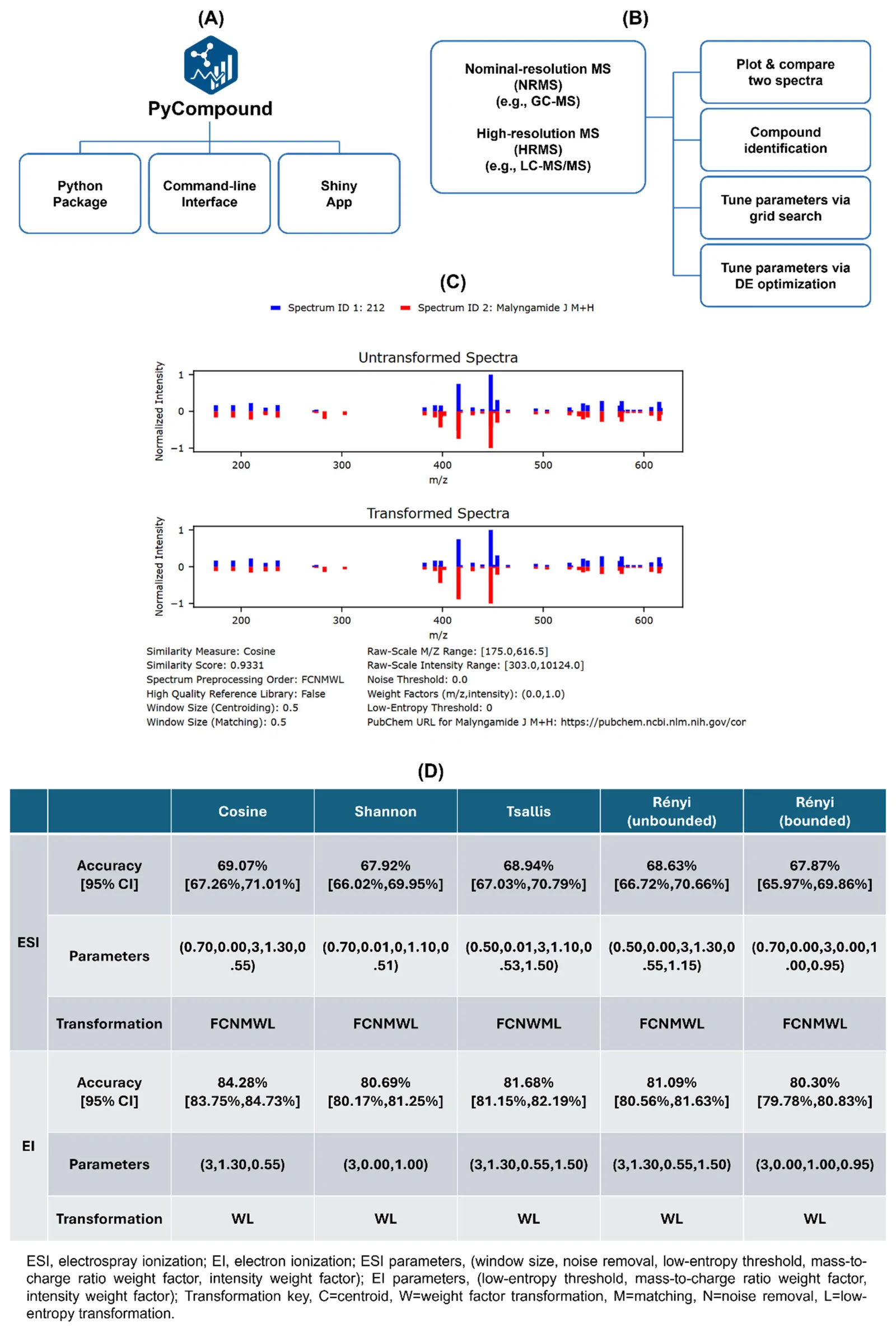
Overview of the PyCompound software design and application. (**A**) Software interfaces implemented in PyCompound, including the Python application programming interface (API), command-line interface (CLI), and interactive Shiny application. (**B**) Schematic overview of the PyCompound workflow for spectral-library matching, including spectral preprocessing, parameter optimization using grid search or differential evolution (DE), similarity calculation, and compound annotation. (**C**) Representative mirror plot comparing a query spectrum (blue) with a reference spectrum (red) following preprocessing, illustrating spectral-library matching for compound annotation. (**D**) Application to the GNPS LC-MS/MS and WebNIST GC-MS datasets showing top-1 annotation accuracies with bootstrap-based 95% confidence intervals, optimized preprocessing workflows and parameter settings, and the performance of cosine, Shannon entropy, Tsallis entropy, and the newly implemented Rényi entropy similarity measures. Rényi (unbounded) denotes the original analysis in which the entropy dimension α was optimized without restriction to 0<α<1, whereas Rényi (bounded) denotes the revised analysis in which α was restricted to 0<α<1, the range over which the proposed Rényi similarity score is theoretically shown to be bounded.

Figure 1A–C provides an overview of the PyCompound architecture, user interfaces, and analytical workflow. PyCompound is implemented in Python 3.12.4 using a modular architecture that separates spectrum preprocessing, similarity calculation, parameter optimization, visualization, and data management into independent components. A distinguishing design feature is the explicit representation of preprocessing order within the analysis workflow, allowing users to define preprocessing sequences according to their analytical requirements. This design is motivated by previous studies demonstrating that preprocessing order can substantially influence spectral-library matching performance [6]. PyCompound also supports user-defined composite similarity measures and automated tuning of preprocessing and similarity parameters to reflect experimental characteristics and analytical configurations. PyCompound supports both nominal-resolution mass spectrometry (NRMS), such as GC-MS, and high-resolution mass spectrometry (HRMS), such as LC-MS/MS, and accepts commonly used spectral formats including MGF, mzML, MSP, CDF, JSON, and tabular text files.

The software is accessible through a Python application programming interface (API), a command-line interface (CLI), and an interactive Shiny web application. Across these interfaces, PyCompound provides four functional components within a unified spectral-library matching workflow: spectral visualization, compound annotation, parameter tuning using grid search, and parameter tuning using differential evolution (DE). The spectral visualization component enables users to compare query and reference spectra before or after preprocessing to evaluate peak alignment, relative intensities, and overall spectral similarity. The compound annotation component preprocesses query spectra, compares them against a reference library using user-selected similarity measures, and returns ranked candidate matches with corresponding similarity scores. In the Shiny application, the query spectrum, the top matched reference spectrum, and direct PubChem links are displayed when available. The parameter-tuning components use either grid search for discrete parameter combinations or DE optimization for continuous parameters to identify preprocessing and similarity-measure settings that maximize annotation accuracy using labeled query spectra with known compound identities.

A detailed comparison of the functionality of PyCompound and representative existing software packages is provided in Table S1. Detailed descriptions of the user interfaces, required inputs, outputs, and example workflows for each functional component are provided in Table S2 and the Supporting Information S1.

### 2.2. Spectral Preprocessing and Preprocessing Order

Spectral preprocessing reduces noise, standardizes spectra, and enhances informative fragmentation patterns prior to similarity calculation. Because optimal preprocessing depends on the dataset and analytical objective, PyCompound implements six preprocessing procedures: filtering, weight-factor transformation, low-entropy transformation, centroiding, noise removal, and peak matching. These individual procedures are not introduced as new methods in this work. Rather, PyCompound’s contribution lies in its configurable integration, allowing users to select and combine procedures, define their execution order subject to applicable constraints, and optimize their parameters within a unified framework. This functionality enables preprocessing workflows to be tailored to different analytical requirements. All six procedures are available for HRMS data, whereas centroiding and peak matching are generally unnecessary for NRMS data.

**Filtering**: Removes peaks outside specified *m*/*z* or intensity ranges to reduce spectral complexity and retain fragment ions most relevant for compound annotation [7,10].

**Weight-factor transformation**: Adjusts peak intensities using *m*/*z* and intensity exponents [9,20]. Appropriate weighting emphasizes structurally informative or highly reproducible fragment ions and can improve spectral-library matching performance.

**Low-entropy transformation**: Originally developed for entropy-based spectral comparison of spectra dominated by a small number of highly intense peaks [3,7]. The procedure computes spectral entropy and selectively redistributes peak intensities below a specified entropy threshold. PyCompound implements this transformation as an optional preprocessing step for all similarity measures [3,6].

**Centroiding**: Merges neighboring peaks within a specified *m*/*z* window and is primarily applied to HRMS data [7,10]. This procedure reduces spectral complexity while improving alignment between query and reference spectra.

**Noise removal**: Eliminates peaks below a specified fraction of the base-peak intensity, reducing the influence of background noise and improving the stability of similarity calculations [7,10].

**Peak matching**: Aligns fragment ions between query and reference spectra within a specified mass tolerance [7,9]. This step is particularly important for HRMS data, where small differences in measured *m*/*z* values may otherwise reduce similarity scores. Formal definitions and implementation details for centroiding and peak matching, including the inclusive mass-window criterion, the handling of multiple peaks within the applicable windows, and the treatment of empty or degenerate spectra, are provided in the Supporting Information S1.

The mathematical definitions and parameterization of each preprocessing procedure are provided in the Supporting Information S1. The execution order of these preprocessing procedures is configurable by the user. For brevity, the preprocessing procedures are denoted as filtering (F), weight-factor transformation (W), low-entropy transformation (L), centroiding (C), noise removal (N), and peak matching (M), with centroiding and peak matching applicable only to HRMS data. For NRMS data represented as discrete peaks at nominal integer *m*/*z* values, centroiding and tolerance-based peak matching are generally unnecessary. For HRMS data, peak matching (M) must be included, and centroiding (C), if used, must precede peak matching. A preprocessing workflow is represented by the ordered sequence of these operations. For instance, the workflow FWLCNM corresponds to the sequence F → W → L → C → N → M, whereas FLWCMN corresponds to F → L → W → C → M → N. Individual preprocessing procedures may also be effectively disabled through their parameter settings (e.g., setting the *m*/*z* and intensity weighting exponents to 0 and 1, respectively, disables weight-factor transformation, whereas setting the low-entropy threshold to 0 disables low-entropy transformation). This flexible representation enables users to investigate the effects of preprocessing order and optimize preprocessing workflows for different datasets and analytical objectives.

### 2.3. Similarity Measures

PyCompound implements nineteen similarity measures, including four continuous measures and fifteen binary measures [6,21]. Let $I_{Q}=\left(a_{1},a_{2},\ldots ,a_{n}\right)$ and $I_{R}=\left(b_{1},b_{2},\ldots ,b_{n}\right)$ denote the normalized intensity vectors of the aligned query and reference spectra, respectively, where

$$a_{i},b_{i}\geq 0,\sum_{i=1}^{n}a_{i}=\sum_{i=1}^{n}b_{i}=1.$$

Thus, each spectrum represents a discrete probability distribution over aligned fragment ions. PyCompound implements four continuous similarity measures: cosine similarity, Shannon entropy similarity, Tsallis entropy similarity, and the newly developed Rényi entropy similarity. The principal definitions are summarized below, whereas complete derivations, normalization procedures, theoretical properties, and proofs are provided in the Supporting Information S1.

### 2.4. Cosine Similarity

Cosine similarity measures the agreement between aligned intensity vectors and is defined as

$$S_{cos}\left(I_{Q},I_{R}\right)=\frac{\sum_{i=1}^{n}a_{i}b_{i}}{\sqrt{\sum_{i=1}^{n}a_{i}^{2}}\sqrt{\sum_{i=1}^{n}b_{i}^{2}}}.$$

Cosine similarity remains the most broadly adopted spectral similarity measure because of its computational efficiency, robustness to overall intensity scaling, and consistently strong empirical performance [4,9].

### 2.5. Shannon Entropy Similarity

Shannon entropy similarity compares spectra as probability distributions [3,6,7]. The Shannon entropy of spectrum I is

$$H_{Shannon}\left(I\right)=-\sum_{i=1}^{n}a_{i}\ln\left(a_{i}\right).$$

Following the generalized Jensen–Shannon divergence [22], the Shannon entropy similarity measure is defined as

$$S_{Shannon}\left(I_{Q},I_{R}\right)=1-\frac{J_{Shannon}\left(I_{Q},I_{R}\right)}{\ln\left(4\right)},$$

where

$$J_{Shannon}\left(I_{Q},I_{R}\right)=2H_{Shannon}\left(\frac{I_{Q}+I_{R}}{2}\right)-H_{Shannon}\left(I_{Q}\right)-H_{Shannon}\left(I_{R}\right).$$

This formulation evaluates similarity according to differences in fragment-ion intensity distributions rather than only direct peak-by-peak agreement.

### 2.6. Tsallis Entropy Similarity

Tsallis entropy introduces an entropy-order parameter q, allowing the relative influence of low- and high-intensity fragment ions to be adjusted [6]. The Tsallis entropy is

$$H_{Tsallis}\left(I,q\right)=\frac{1-\sum_{i=1}^{n}a_{i}^{q}}{q-1},q>0,q\neq 1.$$

The corresponding similarity measure is

$$S_{Tsallis}\left(I_{Q},I_{R},q\right)=1-\frac{J_{Tsallis}\left(I_{Q},I_{R},q\right)}{N_{Tsallis}\left(I_{Q},I_{R},q\right)},$$

where

$$J_{Tsallis}\left(I_{Q},I_{R},q\right)=2H_{Tsallis}\left(\frac{I_{Q}+I_{R}}{2},q\right)-H_{Tsallis}\left(I_{Q},q\right)-H_{Tsallis}\left(I_{R},q\right).$$

As q→1, Tsallis entropy converges to Shannon entropy. The normalization factor $N_{Tsallis}$ is given in the Supporting Information S1.

### 2.7. Rényi Entropy Similarity

PyCompound additionally implements a novel Rényi entropy similarity developed in this work for spectral-library matching. Although Rényi entropy is a well-established concept, it differs from Tsallis entropy in its logarithmic formulation and its additivity for independent probability distributions. In the proposed similarity measure, this formulation provides an alternative scaling of spectral complexity and fragment-intensity distributions relative to Shannon- and Tsallis-based similarities. The Rényi measure is therefore introduced as a mathematically distinct and configurable alternative, rather than as a universally superior similarity measure. Rényi entropy is defined as

$$H_{Renyi}\left(I,q\right)=\frac{1}{1-q}\ln\left(\sum_{i=1}^{n}a_{i}^{q}\right),q>0,q\neq 1.$$

The corresponding similarity measure is

$$S_{Renyi}\left(I_{Q},I_{R},q\right)=1-\frac{J_{Renyi}\left(I_{Q},I_{R},q\right)}{N_{Renyi}\left(I_{Q},I_{R},q\right)},$$

where

$$J_{Renyi}\left(I_{Q},I_{R},q\right)=2H_{Renyi}\left(\frac{I_{Q}+I_{R}}{2},q\right)-H_{Renyi}\left(I_{Q},q\right)-H_{Renyi}\left(I_{R},q\right).$$

For 0<q<1, the similarity is bounded between 0 and 1, whereas this boundedness is not guaranteed for q>1. Complete derivations, normalization procedures, and proofs are provided in the Supporting Information S1.

### 2.8. Binary and Composite Similarity Measures

In addition to continuous measures, PyCompound implements fifteen binary similarity measures, including Jaccard, Dice, Simpson, Kulczynski, Braun-Blanquet, Mountford, and Hamming coefficients [21]. These methods compare spectra using peak presence or absence rather than relative intensity. PyCompound also supports composite similarity measures formed as weighted combinations of two or more implemented similarity measures. Suppose K  similarity measures are selected from the collection implemented in PyCompound. The composite similarity measure is then defined as

$$S_{mixture}\left(I,J\right)=\sum_{k=1}^{K}w_{k}S_{k}\left(I,J\right),$$

where $S_{k}(I,J)$ is the similarity score from the k-th similarity measure and $w_{k}$ is its user-specified weight. Users may specify a nonnegative weight for each implemented similarity measure, with a weight of zero excluding the corresponding measure from the composite score. Before the composite score is calculated, the user-specified weights are normalized so that they sum to one. At least one weight must therefore be greater than zero. By default, equal weights are assigned to the four continuous similarity measures, consisting of cosine, Shannon entropy, Rényi entropy, and Tsallis entropy similarities, while weights of zero are assigned to the fifteen binary measures. Before combination, all component similarity scores are expressed on a common scale from 0 to 1. This includes measures whose original formulations require conversion to this range, such as the Fager-McGowan measure. For Rényi entropy similarity, the score is bounded between 0 and 1 when the entropy-order parameter is restricted to 0<α<1. Because the component scores used in the composite measure are expressed on the same scale, PyCompound combines them directly using the normalized weights and does not perform any additional measure-specific rescaling during calculation of the composite score.

### 2.9. Parameter Optimization

PyCompound provides automated parameter tuning for preprocessing procedures, similarity measures, and reference-library filtering using query spectra with known compound identities. Two optimization strategies are implemented: grid search and DE, a population-based metaheuristic algorithm [23,24]. Users may tune preprocessing and similarity-measure parameters individually or jointly to maximize compound annotation accuracy. These optimization procedures facilitate adaptation of spectral-library matching workflows to different datasets and analytical objectives.

### 2.10. Application: GNPS and WebNIST Datasets

To demonstrate the implementation of PyCompound, we analyzed two publicly available datasets previously examined in our methodological comparison study of continuous similarity measures [6,21]. The WebNIST GC-MS dataset was also previously used to evaluate differential evolution for parameter optimization [23]. Reusing these datasets provides established benchmarks for demonstrating PyCompound’s functionality and enables direct comparison with the previously evaluated methods. Because the Rényi entropy similarity is newly introduced in PyCompound, this application focuses on comparing its annotation performance with those of the previously implemented continuous similarity measures. The datasets represent two widely used mass spectrometry platforms in metabolomics, encompassing both high-resolution (LC-MS/MS) and nominal-resolution (GC-MS) workflows. The first dataset consisted of electrospray ionization (ESI)-based LC-MS/MS spectra from the Global Natural Products Social Molecular Networking (GNPS) platform, whereas the second consisted of electron-ionization (EI)-based GC-MS spectra from the WebNIST database. The construction, selection, and preprocessing of these benchmark datasets were described previously [21] and the principal characteristics relevant to the present analysis are summarized below.

For the WebNIST GC-MS benchmark, EI mass spectra extracted from the NIST Chemistry WebBook on 28 November 2011 served as the reference library, and replicate spectra from the NIST 08 Mass Spectral Library served as the query data. The original reference library contained 23,721 spectra representing 23,721 unique compounds, whereas the original replicate library contained 28,307 spectra representing 18,569 unique compounds. Compounds not represented in the reference library were removed from the query data, leaving 21,516 query spectra representing 12,850 unique compounds. Thus, the final reference library contained 23,721 entries representing 23,721 unique compounds, and all compounds retained in the query dataset were represented in the reference library. The spectra contained *m*/*z* values ranging from 1 to 892.

For the GNPS LC-MS/MS benchmark, ESI mass spectra were extracted from GNPS on 2 February 2022. From the 44 available GNPS libraries, which collectively contained 481,093 spectra representing 150,798 unique compounds, 22 libraries were selected, and the data were restricted to high-quality spectra acquired in positive-ion mode, yielding 34,718 spectra representing 25,576 unique compounds. Spectra were limited to *m*/*z* ≤ 1000, peaks with relative intensities below 0.01 of the maximum peak intensity were removed, and spectra containing fewer than 10 remaining peaks were excluded. After preprocessing, 17,103 spectra representing 14,705 unique compounds remained. The reference library was constructed using the single available spectrum for each of 13,458 compounds and one randomly selected spectrum for each of the 1247 compounds having replicate spectra, resulting in 14,705 reference spectra representing 14,705 unique compounds. The remaining replicate spectra formed the query dataset, which contained 2398 spectra representing 1247 unique compounds. The resulting spectra contained *m*/*z* values ranging from 100 to 1000.

For both benchmarks, the reference library contained one mass spectrum per compound and therefore had no duplicate entries at the compound level. In contrast, the query datasets intentionally retained replicate spectra for the same compound. Each replicate spectrum was treated as an independent query, compared against the complete reference library, and included separately in the spectrum-level calculation of annotation accuracy. For the GNPS benchmark, one spectrum from each compound having replicate spectra was randomly assigned to the reference library, and the remaining spectra were used as queries. Therefore, the same spectral record was not included in both sets. For the WebNIST benchmark, the reference and query spectra originated from separate sources: the NIST Chemistry WebBook and the NIST 08 replicate library, respectively. Reference-library entries were ranked in descending order of similarity score. When similarity scores were tied, the first tied entry in the existing reference-library order was selected, and ties were not treated as co-ranked.

A query spectrum was considered correctly annotated at rank n if the reference-library entry corresponding to the same compound appeared among the n entries with the highest similarity scores. Accordingly, top-1, top-5, and top-10 annotation accuracies were calculated as the proportions of query spectra for which the correct compound occurred among the first 1, 5, and 10 ranked reference-library entries, respectively.

Consistent with the procedure used in Ref. [6], 95% confidence intervals for annotation accuracy were calculated using 1000 bootstrap replicates sampled with replacement. The selected preprocessing workflow and similarity-specific parameter settings were held fixed during bootstrap resampling and were not retuned within individual bootstrap replicates. The same random seed was used across method comparisons under each experimental condition so that the methods were evaluated using the same bootstrap samples.

Following the procedure established previously [6], multiple preprocessing orders and similarity-specific parameter combinations were evaluated using the labeled query spectra, and the configuration achieving the highest annotation accuracy was selected for each similarity measure. The selected configuration was subsequently used to calculate top-1, top-5, and top-10 annotation accuracies with bootstrap-based 95% confidence intervals on the same query dataset. Independent test data and nested cross-validation were not used in the present application. Accordingly, the reported results characterize performance under optimized conditions on these demonstration datasets and should not be interpreted as independent estimates of generalization to unseen data.

Figure 1D compares the top-1 annotation performance of cosine similarity, Shannon entropy similarity, Tsallis entropy similarity, and both the unbounded and bounded Rényi entropy similarity analyses under optimized conditions. “Rényi (unbounded)” denotes the original analysis in which the entropy dimension α was optimized without restriction to 0<α<1, whereas “Rényi (bounded)” denotes the revised analysis in which α was restricted to 0<α<1, the range over which the proposed Rényi similarity score is theoretically shown to be bounded. The bounded Rényi analysis achieved top-1 annotation accuracies of 80.30% for the WebNIST GC-MS dataset and 67.87% for the GNPS LC-MS/MS dataset. These results were comparable to those obtained with the established Shannon and Tsallis entropy similarity measures across both datasets. Consistent with our previous methodological study, cosine similarity achieved the highest top-1 annotation accuracy for both datasets. Detailed top-1 annotation accuracies, optimized preprocessing workflows, and parameter settings for both Rényi analyses are provided in Tables S3 and S4. Figures S1 and S2 of Supporting Information S1 present annotation performance across candidate ranks for the original unbounded Rényi analysis but do not include the bounded Rényi analysis. Complete numerical results for top-1, top-5, and top-10 annotation accuracies and their corresponding bootstrap-based 95% confidence intervals for both Rényi analyses are provided in Supporting Information S2. The original unbounded results are retained for transparency and comparison, whereas the principal interpretation of Rényi similarity is based on the bounded analysis.

## 3. Conclusions

PyCompound is an open-source Python package for spectral-library matching in metabolomics that integrates flexible preprocessing workflows, nineteen continuous and binary similarity measures, user-defined composite similarity scores, and automated parameter optimization within a unified framework supporting both nominal-resolution and high-resolution mass spectrometry data. The software is accessible through a Python API, a command-line interface, and an interactive Shiny application, facilitating its use across diverse analytical workflows. Application to the public GNPS LC-MS/MS and WebNIST GC-MS datasets demonstrates the implementation of PyCompound for spectral-library matching and evaluates the performance of the newly developed Rényi entropy similarity relative to established similarity measures. In both datasets, the theoretically bounded analysis (0<α<1) and the unrestricted analysis demonstrated that Rényi entropy similarity achieved annotation performance comparable to that of the established Shannon and Tsallis entropy similarity measures.

Several limitations of the present demonstration should be noted. As with any spectral-library-matching approach, annotation is limited by the coverage and quality of the reference library, and compounds absent from the library cannot be directly annotated. Moreover, evaluation using the two datasets does not encompass the full diversity of mass spectrometry instruments, acquisition conditions, and sample types. Additional independent datasets or nested validation will therefore be valuable for obtaining less biased estimates of generalization performance across analytical settings. Although routine spectral-library matching does not require labeled training data, labeled query datasets with known compound identities are needed to evaluate annotation performance and objectively optimize preprocessing workflows and similarity-specific parameters. Because the same labeled query datasets were used for both parameter selection and performance evaluation, the reported accuracies describe performance under optimized conditions and may be optimistic relative to performance on independent data. The present application demonstrates configurable preprocessing, similarity comparison, and parameter optimization within PyCompound but does not establish that these capabilities universally improve annotation performance. Controlled comparisons of default and optimized configurations across additional datasets are needed to determine when and to what extent these capabilities improve performance. The current implementation also focuses on similarity-based spectral-library matching and does not incorporate complementary annotation evidence, such as retention time, collision cross section, or machine-learning-based structure prediction. Consequently, spectral similarity alone should not be interpreted as providing an unambiguous structural identification, particularly for isomeric candidates.

PyCompound is intended as a standalone and configurable tool for the spectral-library-matching component of a broader compound-annotation workflow. Its modular implementation as a Python package allows it to be incorporated into computational workflows or used together with existing Python packages that evaluate complementary annotation evidence. Direct integration of such evidence into PyCompound is planned for future releases. By providing a flexible, extensible, and reproducible platform for spectral-library matching, PyCompound serves as a practical resource for compound annotation and future methodological development in metabolomics and small-molecule mass spectrometry.

## Data Availability

PyCompound is freely available under an open-source MIT license at https://github.com/hdlugas/pycompound (accessed on 14 June 2026) and via PyPI (https://pypi.org/project/pycompound/, accessed on 14 June 2026). The GitHub repository provides the dependency versions, parameter grids, optimization settings, analysis scripts, and configuration files required to reproduce the reported results. An interactive Shiny application (https://connect.posit.cloud/fy7392, accessed on 14 June 2026), video tutorials (https://www.youtube.com/@PyCompound, accessed on 14 June 2026), and the reference libraries and validation datasets used in this study (https://zenodo.org/records/12786324, accessed on 14 June 2026) are also provided.

## Supplementary Materials

The following supporting information can be downloaded at: https://www.mdpi.com/article/10.3390/metabo16080585/s1, The Supporting Information S1 provides supplementary methodological details, mathematical derivations and proofs, a comparison of PyCompound with representative existing software packages (Table S1), descriptions of the user interfaces and analysis modes (Table S2), annotation accuracy and optimized parameter settings for the GNPS and WebNIST datasets (Tables S3 and S4), and additional figures (Figures S1 and S2). Supporting Information S2 provides complete numerical results for top-1, top-5, and top-10 annotation accuracies and their corresponding bootstrap-based 95% confidence interval.
