# Supplementary material for: PyCompound: An Open-Source Python Package for Spectral-Library Matching in Mass Spectrometry-Based Metabolomics
Source: Metabolites. 2026 Aug 18;16(8):585. doi: 10.3390/metabo16080585 (PMC13515664; doi:10.3390/metabo16080585)
Supplement: Supplementary file 1 [file metabolites-16-00585-s001.zip › PyCompound_Revision_Supporting Information S1_08102026.pdf]

Supporting Information

PyCompound: An Open-Source Python Package for Spectral-Library Matching in Mass Spectrometry-Based Metabolomics

Hunter Dlugas[1], Xiang Zhang[2], Xun Bao[3,4], Jing Li[3,4], Ikuko Kato[5], and Seongho Kim[1,3,*]

[1] Biostatistics and Bioinformatics Core, Karmanos Cancer Institute, Wayne State University, Detroit, MI
[2] Department of Chemistry, University of Louisville, Louisville, KY
[3] Department of Oncology, School of Medicine, Wayne State University, Detroit, MI
[4] Pharmacology and Metabolomics Core, Karmanos Cancer Institute, Wayne State University, Detroit, MI
[5] Epidemiology Division, Populations Sciences in the Pacific Program, University of Hawaii Cancer Center, Honolulu, HI

[*] To whom correspondence should be addressed: `kimse@karmanos.org`

Mass Spectrum Preprocessing

We consider several procedures involved in mass spectrum preprocessing, each representing a type of mass spectrum transformation. These procedures include filtering, weight-factor transformation, low-entropy transformation, centroiding, noise removal, and matching. In the `PyCompound` package, they are abbreviated using single-letter codes: F, W, L, C, N, and M, respectively. Among these six procedures, centroiding and matching apply only to high-resolution mass spectrometry (HRMS) data.

Let $z = (z_1, z_2, \ldots, z_n)$ denote the ordered m/z values of a mass spectrum of length n. We represent the corresponding intensity vector by $I = (x_1, x_2, \ldots, x_n)$, where each intensity satisfies $x_i \geq 0$. Thus, a mass spectrum is characterized by the m/z vector z and the intensity vector I.

- **Filtering (F):** Given user-defined parameters $(z_{\min}, z_{\max})$ and $(x_{\min}, x_{\max})$ and a spectrum I, the transformed spectrum $I^* = (x_1^*, x_2^*, \ldots, x_n^*)$ is defined by

$$x_i^* = \begin{cases} x_i, & \text{if } z_{\min} \leq z_i \leq z_{\max} \text{ and } x_{\min} \leq x_i \leq x_{\max}, \\ 0, & \text{otherwise.} \end{cases}$$

- **Weight-factor transformation (W):** Given user-defined parameters (α, β) and a spectrum I, the transformed spectrum I^* has the same m/z values as I and intensities

$$I^* = \left(z_1^\alpha x_1^\beta,\, z_2^\alpha x_2^\beta,\, \ldots,\, z_n^\alpha x_n^\beta\right).$$

- **Low-entropy transformation (L):** Given a user-defined low-entropy threshold T and a spectrum I, define

$$a_i = \frac{x_i}{\sum_{j=1}^{n} x_j}, \qquad i = 1, \ldots, n,$$

and the Shannon entropy

$$H_{Shannon}(I) = -\sum_{i=1}^{n} a_i \ln(a_i).$$

The transformed intensities $I^* = (x_1^*, x_2^*, \ldots, x_n^*)$ are given by

$$x_i^* = \begin{cases} a_i, & \text{if } H_{Shannon}(I) \geq T, \\ a_i^{\frac{1+H_{Shannon}(I)}{1+T}}, & \text{if } H_{Shannon}(I) < T, \end{cases} \qquad i = 1, \ldots, n.$$

- **Centroiding (C; HRMS only):** Given a user-defined window-size parameter $w_{\text{centroiding}}$ and a spectrum I, the transformed spectrum $I^\star$ merges adjacent peaks (z_i, x_i) and (z_{i+1}, x_{i+1}) into a single peak

$$\left(\frac{z_i x_i + z_{i+1} x_{i+1}}{x_i + x_{i+1}},\ x_i + x_{i+1} \right)$$

whenever $|z_i - z_{i+1}| \leq w_{\text{centroiding}}$ for $i \in \{1, 2, \ldots, n-1\}$. This centroiding procedure generalizes naturally to cases where more than two peaks have m/z values within $w_{\text{centroiding}}$ of one another.

- **Noise removal (N):** Given a user-defined parameter r and a spectrum I, this procedure removes all peaks whose intensities satisfy

$$x_j < r \cdot \max\{x_1, x_2, \ldots, x_n\}, \qquad j \in \{1, 2, \ldots, n\}.$$

- **Matching (M; HRMS only):** Given a user-defined window-size parameter w_{matching} and two spectra with intensities $I = (x_1, x_2, \ldots, x_n)$ and $J = (y_1, y_2, \ldots, y_m)$, and corresponding m/z values $(z_1, z_2, \ldots, z_n)$ and $(v_1, v_2, \ldots, v_m)$, the goal is to produce two spectra $I^\star$ and $J^\star$ that contain the same number of peaks and share identical m/z values. For each peak (z_i, x_i) in I:
  - If there is no peak (v_j, y_j) in J satisfying $|z_i - v_j| \leq w_{\text{matching}}$, then (z_i, x_i) is included in $I^\star$ and the peak $(z_i, 0)$ is included in $J^\star$.
  - If there is at least one peak (v_j, y_j) in J satisfying $|z_i - v_j| \leq w_{\text{matching}}$, then (z_i, x_i) is included in $I^\star$ and the peak

    $$(z_i, \sum_{j:\, |z_i - v_j| < w_{\text{matching}}} y_j)$$

    is included in $J^\star$.

  The same procedure is then applied after swapping the roles of I and J.

Implementation details for centroiding and peak matching. In PyCompound, centroiding uses a greedy, intensity-priority procedure. Peaks are first sorted by m/z and then processed from highest to lowest intensity. For each peak with positive intensity, neighboring peaks satisfying

$$|\Delta m/z| \leq w_{\text{centroiding}}$$

are merged. The m/z value of the resulting peak is calculated as the intensity-weighted mean, and its intensity is calculated as the sum of the merged intensities. Merged peaks are assigned zero intensity so that they are not reused. If no centroiding is required, the original spectrum is retained. If centroiding leaves fewer than two peaks, the implementation returns the placeholder spectrum $\big[[0,0]\big]$.

Peak matching aligns the query and reference spectra through a two-pointer merge of peaks sorted by m/z. Peaks satisfying

$$|\Delta m/z| \leq w_{\text{matching}}$$

are matched, whereas peaks outside the matching window are treated as unmatched and assigned an intensity of zero in the other spectrum. When multiple peaks fall within the same matching window, their intensities are summed according to the implemented matching procedure. The resulting aligned representation contains three columns: m/z, query-spectrum intensity, and reference-spectrum intensity. If no aligned rows are produced, the implementation returns the placeholder row $\big[[0,0,0]\big]$.

Within the spectral-library matching workflow, infinite intensity values generated during transformation are reset to zero. Centroiding, peak matching, and subsequent transformations are performed only when both spectra contain more than one peak. If either spectrum contains fewer than two peaks after preprocessing or either aligned intensity vector has a total intensity of zero, the similarity score is set to zero. Thus, these empty or degenerate spectra do not produce undefined similarity values.

These preprocessing steps are also documented in the `PyCompound` GitHub repository (`https://github.com/hdlugas/pycompound`).

Boundedness of entropy-based similarity measures

We denote the spectra whose similarity we wish to compute by $I_Q = (a_1, a_2, \ldots, a_n)$ and $I_R = (b_1, b_2, \ldots, b_n)$, where $\sum_{i=1}^{n} a_i = \sum_{i=1}^{n} b_i = 1$ and $0 \leq a_i, b_i \leq 1$ for all i. Each spectrum therefore represents a discrete probability distribution. All logarithms denote the natural logarithm.

Within this probabilistic framework, the generalized Jensen–Shannon divergence [3], $JS_\pi(I_1, \ldots, I_N)$, quantifies dissimilarity among N probability distributions $I_1, \ldots, I_N$ with weights $\pi = (\pi_1, \ldots, \pi_N)$:

$$JS_\pi(I_1, \ldots, I_N) = H_{Shannon}\left(\sum_{i=1}^{N} \pi_i I_i\right) - \sum_{i=1}^{N} \pi_i \, H_{Shannon}(I_i),$$

where $H_{Shannon}(I) = -\sum_{i=1}^{n} a_i \ln(a_i)$ is the Shannon entropy of $I = (a_1, \ldots, a_n)$. All three entropy-based similarity measures (Shannon, Tsallis, and Rényi) in this work arise from the special case $N = 2$ with $\pi = (1/2, 1/2)$. The Tsallis and Rényi versions are obtained by replacing the Shannon entropy in this expression with the Tsallis or Rényi entropy, respectively. Note that the Shannon-based and Tsallis-based measures were introduced in [2] and [1], respectively, whereas the Rényi-based measure is newly introduced in this work.

In what follows, we show that the three entropy-based similarity measures used in this work are bounded between 0 and 1. A value of 1 occurs exactly when the query and reference spectra are identical, and a value of 0 occurs when they have disjoint support, meaning that the spectra share no m/z values with nonzero intensity in common. For the Tsallis and Rényi measures, the parameter q denotes the usual entropy order appearing in their definitions. In the Rényi case, boundedness in $[0, 1]$ holds when $q \in (0, 1)$, whereas the Tsallis-based measure is bounded in $[0, 1]$ for all $q > 0$, $q \neq 1$.

Shannon

The Shannon entropy of a probability distribution $I = (a_1, \ldots, a_n)$ is

$$H_{Shannon}(I) = -\sum_{i=1}^{n} a_i \ln(a_i).$$

To verify concavity, define $f(x) = x \ln(x)$ for $x > 0$ and set $f(0) = 0$ to ensure continuity on $[0, \infty)$. Since f is convex, the function $-x \ln(x)$ is concave, and therefore $H_{Shannon}$ is concave on the space of probability distributions.

Definition 1 ([2]). The Shannon entropy similarity measure between spectra $I_Q = (a_1, \ldots, a_n)$ and $I_R = (b_1, \ldots, b_n)$ is

$$S_{Shannon}(I_Q, I_R) = 1 - \frac{J_{Shannon}(I_Q, I_R)}{\ln(4)},$$

where

$$\begin{aligned} J_{Shannon}(I_Q, I_R) &= 2\, JS_\pi(I_Q, I_R) \\ &= 2\, H_{Shannon}\left(\frac{I_Q + I_R}{2}\right) - H_{Shannon}(I_Q) - H_{Shannon}(I_R), \end{aligned}$$

and $\pi = (1/2, 1/2)$.

We now establish its basic boundedness properties.

Theorem 1. The Shannon entropy similarity measure $S_{Shannon}(I_Q, I_R)$ lies in $[0, 1]$. Furthermore, $S_{Shannon}(I_Q, I_R) = 1$ if and only if $I_Q = I_R$, and $S_{Shannon}(I_Q, I_R) = 0$ if and only if I_Q and I_R have disjoint support (i.e. $a_i b_i = 0$ for all i).

Proof. Since $H_{Shannon}$ is concave, Jensen's inequality gives

$$H_{Shannon}\left(\frac{I_Q + I_R}{2}\right) \geq \frac{1}{2}H_{Shannon}(I_Q) + \frac{1}{2}H_{Shannon}(I_R),$$

implying $J_{Shannon}(I_Q, I_R) \geq 0$ and therefore $S_{Shannon}(I_Q, I_R) \leq 1$. If $I_Q = I_R$, then $J_{Shannon}(I_Q, I_R) = 0$, so $S_{Shannon}(I_Q, I_Q) = 1$.

To show $S_{Shannon}(I_Q, I_R) \geq 0$, it suffices to prove that $J_{Shannon}(I_Q, I_R) \leq \ln(4)$. A direct computation gives

$$\begin{aligned} J_{Shannon}(I_Q, I_R) &= \sum_{i=1}^{n}\left(a_i \ln\left(\frac{a_i}{a_i + b_i}\right) + b_i \ln\left(\frac{b_i}{a_i + b_i}\right) + (a_i + b_i)\ln(2)\right) \\ &= \sum_{i=1}^{n} X_i + \ln(4), \end{aligned}$$

where

$$X_i = a_i \ln\left(\frac{a_i}{a_i + b_i}\right) + b_i \ln\left(\frac{b_i}{a_i + b_i}\right).$$

Since $\frac{a_i}{a_i+b_i}, \frac{b_i}{a_i+b_i} \in (0, 1]$ and $\ln(x) \leq 0$ for $x \in (0, 1]$, and adopting the convention $0 \ln 0 = 0$, each $X_i \leq 0$. Hence $\sum_{i=1}^{n} X_i \leq 0$, which implies $J_{Shannon}(I_Q, I_R) \leq \ln(4)$ and therefore $S_{Shannon}(I_Q, I_R) \geq 0$.

Equality $J_{Shannon}(I_Q, I_R) = \ln(4)$ holds exactly when all $X_i = 0$, which occurs if and only if $a_i b_i = 0$ for every i. This corresponds to disjoint support, and yields $S_{Shannon}(I_Q, I_R) = 0$. This completes the proof. □

Tsallis

The Tsallis entropy of a probability distribution $I = (a_1, \ldots, a_n)$ is defined by

$$H_{Tsallis}(I, q) = \frac{1 - \sum_{i=1}^{n} a_i^q}{q - 1}, \qquad q > 0,\ q \neq 1.$$

As $q \to 1$, $H_{Tsallis}(I, q)$ converges to the Shannon entropy $H_{Shannon}(I)$. For each fixed $q > 0$, $q \neq 1$, $H_{Tsallis}(I, q)$ is concave on the space of probability distributions. Let $\lambda \in (0, 1)$. The concavity inequality

$$H_{Tsallis}(\lambda I_Q + (1 - \lambda) I_R, q) \geq \lambda H_{Tsallis}(I_Q, q) + (1 - \lambda) H_{Tsallis}(I_R, q)$$

is equivalent to

$$\begin{aligned} \sum_{i=1}^{n}(\lambda a_i + (1 - \lambda) b_i)^q &\geq \lambda \sum_{i=1}^{n} a_i^q + (1 - \lambda) \sum_{i=1}^{n} b_i^q \quad \text{for } q \in (0, 1), \\ \sum_{i=1}^{n}(\lambda a_i + (1 - \lambda) b_i)^q &\leq \lambda \sum_{i=1}^{n} a_i^q + (1 - \lambda) \sum_{i=1}^{n} b_i^q \quad \text{for } q > 1. \end{aligned}$$

These inequalities follow from applying Jensen's inequality to the function x^q, which is concave on $[0,1]$ for $q \in (0,1)$ and convex on $[0,1]$ for $q > 1$, in each coordinate and then summing over i.

The Tsallis entropy–based similarity measure, in direct analogy with the Shannon case, is defined as follows.

Definition 2 ([1])**.** For $q > 0$, $q \neq 1$, the Tsallis entropy similarity measure between spectra $I_Q = (a_1, \dots, a_n)$ and $I_R = (b_1, \dots, b_n)$ is

$$S_{Tsallis}(I_Q, I_R, q) = 1 - \frac{J_{Tsallis}(I_Q, I_R, q)}{N_{Tsallis}(I_Q, I_R, q)},$$

where

$$J_{Tsallis}(I_Q, I_R, q) = 2\, H_{Tsallis}\left(\frac{I_Q + I_R}{2}, q\right) - H_{Tsallis}(I_Q, q) - H_{Tsallis}(I_R, q)$$

and

$$N_{Tsallis}(I_Q, I_R, q) = \frac{\sum_{i=1}^{n} \left(2\left(\frac{a_i}{2}\right)^q + 2\left(\frac{b_i}{2}\right)^q - a_i^q - b_i^q\right)}{1-q}.$$

The quantity $N_{Tsallis}(I_Q, I_R, q)$ is always positive. Indeed,

$$2\left(\frac{a_i}{2}\right)^q = 2^{\,1-q} a_i^q, \qquad 2\left(\frac{b_i}{2}\right)^q = 2^{\,1-q} b_i^q,$$

so

$$N_{Tsallis}(I_Q, I_R, q) = \frac{\left(2^{1-q} - 1\right)\left(\sum_{i=1}^{n} a_i^q + \sum_{i=1}^{n} b_i^q\right)}{1-q}.$$

The factor $\sum_{i=1}^{n} a_i^q + \sum_{i=1}^{n} b_i^q$ is strictly positive. For $q \in (0,1)$ we have $2^{1-q} > 1$ and $1 - q > 0$, so the quotient is positive. For $q > 1$ we have $2^{1-q} < 1$ and $1 - q < 0$, so numerator and denominator are both negative and the quotient is again positive.

Theorem 2. For every $q > 0$, $q \neq 1$, the Tsallis entropy similarity measure $S_{Tsallis}(I_Q, I_R, q)$ lies in $[0,1]$. Moreover, $S_{Tsallis}(I_Q, I_R, q) = 1$ if and only if $I_Q = I_R$, and $S_{Tsallis}(I_Q, I_R, q) = 0$ if and only if I_Q and I_R have disjoint support (i.e. $a_i b_i = 0$ for all i).

Proof. Since $H_{Tsallis}(I, q)$ is concave for every $q > 0$, $q \neq 1$, Jensen's inequality gives

$$H_{Tsallis}\left(\frac{I_Q + I_R}{2}, q\right) \geq \frac{1}{2} H_{Tsallis}(I_Q, q) + \frac{1}{2} H_{Tsallis}(I_R, q),$$

so $J_{Tsallis}(I_Q, I_R, q) \geq 0$. Since $N_{Tsallis}(I_Q, I_R, q) > 0$, this implies

$$S_{Tsallis}(I_Q, I_R, q) = 1 - \frac{J_{Tsallis}(I_Q, I_R, q)}{N_{Tsallis}(I_Q, I_R, q)} \leq 1.$$

If $I_Q = I_R$, then $(I_Q{+}I_R)/2 = I_Q$, so $J_{Tsallis}(I_Q, I_Q, q) = 0$ and $S_{Tsallis}(I_Q, I_Q, q) = 1$.

To show $S_{Tsallis} \geq 0$, it suffices to prove that $J_{Tsallis}(I_Q, I_R, q) \leq N_{Tsallis}(I_Q, I_R, q)$. A direct calculation shows this is equivalent to

$$\sum_{i=1}^{n} \left(\frac{a_i + b_i}{2}\right)^q \leq \sum_{i=1}^{n} \left(\frac{a_i}{2}\right)^q + \sum_{i=1}^{n} \left(\frac{b_i}{2}\right)^q \qquad \text{for } q \in (0,1),$$

$$\sum_{i=1}^{n} \left(\frac{a_i + b_i}{2}\right)^q \geq \sum_{i=1}^{n} \left(\frac{a_i}{2}\right)^q + \sum_{i=1}^{n} \left(\frac{b_i}{2}\right)^q \qquad \text{for } q > 1.$$

These inequalities follow from the concavity of x^q for $q \in (0,1)$ and its convexity for $q > 1$, applied coordinatewise and summed over i.

Finally, we characterize when $S_{Tsallis}(I_Q, I_R, q) = 0$. By definition, $S_{Tsallis} = 0$ if and only if $J_{Tsallis} = N_{Tsallis}$, which is equivalent to

$$\sum_{i=1}^{n} \left(\frac{a_i + b_i}{2}\right)^q = \sum_{i=1}^{n} \left(\frac{a_i}{2}\right)^q + \sum_{i=1}^{n} \left(\frac{b_i}{2}\right)^q.$$

For $q \in (0,1)$, the function x^q is strictly concave on $[0, \infty)$, so whenever $a_i > 0$ and $b_i > 0$,

$$\left(\frac{a_i + b_i}{2}\right)^q < \left(\frac{a_i}{2}\right)^q + \left(\frac{b_i}{2}\right)^q.$$

For $q > 1$, the function x^q is strictly convex, and for $a_i, b_i > 0$,

$$\left(\frac{a_i + b_i}{2}\right)^q > \left(\frac{a_i}{2}\right)^q + \left(\frac{b_i}{2}\right)^q.$$

In either case, whenever $a_i > 0$ and $b_i > 0$, the i-th term of the left sum is strictly unequal to the corresponding sum of terms on the right. Therefore, equality

$$\sum_{i=1}^{n} \left(\frac{a_i + b_i}{2}\right)^q = \sum_{i=1}^{n} \left(\frac{a_i}{2}\right)^q + \sum_{i=1}^{n} \left(\frac{b_i}{2}\right)^q$$

can hold only if for each index i at least one of a_i or b_i is zero. That is,

$$a_i b_i = 0 \qquad \text{for all } i,$$

which means I_Q and I_R have disjoint support. This completes the proof. $\square$

Rényi

The Rényi entropy of a probability distribution $I = (a_1, \dots, a_n)$ is defined by

$$H_{Rényi}(I, q) = \frac{1}{1-q} \ln\left(\sum_{i=1}^{n} a_i^q\right), \qquad q > 0,\ q \neq 1.$$

As $q \to 1$, $H_{Rényi}(I, q)$ converges to $H_{Shannon}(I)$. For $q \in (0, 1)$, the function a^q is concave on $[0, 1]$, so

$$\sum_{i=1}^{n} a_i^q$$

is concave in I. The function $\ln(x)$ is concave and nondecreasing on $(0, \infty)$, and the composition of a concave, nondecreasing function with a concave function is concave. Thus, for $q \in (0, 1)$, the function

$$\ln\left(\sum_{i=1}^{n} a_i^q\right)$$

is concave, and multiplication by the positive scalar $\frac{1}{1-q}$ preserves concavity. Therefore, $H_{Rényi}(I, q)$ is concave on the space of probability distributions for every $q \in (0, 1)$.

The Rényi entropy–based similarity measure, a novel measure introduced in this work and constructed in direct analogy with the Shannon case, is defined as follows.

Definition 3. For $q > 0$, $q \neq 1$, the Rényi entropy similarity measure between two spectra $I_Q = (a_1, \dots, a_n)$ and $I_R = (b_1, \dots, b_n)$ is

$$S_{Rényi}(I_Q, I_R, q) = 1 - \frac{J_{Rényi}(I_Q, I_R, q)}{N_{Rényi}(I_Q, I_R, q)},$$

where

$$J_{Rényi}(I_Q, I_R, q) = 2\, H_{Rényi}\left(\frac{I_Q + I_R}{2}, q\right) - H_{Rényi}(I_Q, q) - H_{Rényi}(I_R, q)$$

and

$$N_{Rényi}(I_Q, I_R, q) = \left(\frac{1}{1-q}\right) \left[2 \ln\left(\sum_{i=1}^{n} \left(\frac{a_i}{2}\right)^q + \sum_{i=1}^{n} \left(\frac{b_i}{2}\right)^q\right) - \ln\left(\sum_{i=1}^{n} a_i^q\right) - \ln\left(\sum_{i=1}^{n} b_i^q\right)\right].$$

For $q \in (0, 1)$, the normalization factor $N_{Rényi}(I_Q, I_R, q)$ is strictly positive. Let

$$X = \sum_{i=1}^{n} a_i^q, \qquad Y = \sum_{i=1}^{n} b_i^q.$$

Then

$$N_{Rényi}(I_Q, I_R, q) = \frac{1}{1-q}\left(2\ln\bigl(2^{-q}(X+Y)\bigr) - \ln(X) - \ln(Y)\right).$$

By the arithmetic mean–geometric mean inequality, $X + Y \geq 2\sqrt{XY}$. Since $2^{-q} > 0$ and the function $\ln(x)$ is increasing on $(0, \infty)$,

$$\ln\bigl(2^{-q}(X+Y)\bigr) \geq \ln\bigl(2^{-q+1}(XY)^{1/2}\bigr).$$

For $q \in (0,1)$, we have $1 - q > 0$, so

$$\begin{aligned} N_{Rényi}(I_Q, I_R, q) &\geq \frac{1}{1-q}\Bigl(2\ln\bigl(2^{-q+1}(XY)^{1/2}\bigr) - \ln(X) - \ln(Y)\Bigr) \\ &= \frac{1}{1-q}\bigl(2(1-q)\ln 2\bigr) \\ &= \ln(4) > 0. \end{aligned}$$

For $q \in (0,1)$, the Rényi entropy $H_{Rényi}(I, q)$ is concave on the space of probability distributions, and this concavity underlies the boundedness of the resulting similarity measure. In contrast, for $q > 1$, $H_{Rényi}(I, q)$ is no longer concave in general. When $q > 1$, the quantity $J_{Rényi}(I_Q, I_R, q)$ may become negative while the normalization factor $N_{Rényi}(I_Q, I_R, q)$ remains positive, which can produce similarity values exceeding 1.

As an explicit illustration for $q > 1$, consider $q = 2$ and

$$\begin{aligned} I_Q &= (0.77,\ 0.12,\ 0.11), \\ I_R &= (0.99,\ 0.005,\ 0.005). \end{aligned}$$

A direct computation shows

$$\begin{aligned} J_{Rényi}(I_Q, I_R, 2) &= 2\,H_{Rényi}\left(\frac{I_Q + I_R}{2},\ 2\right) - H_{Rényi}(I_Q, 2) - H_{Rényi}(I_R, 2) \\ &< 0. \end{aligned}$$

Since $N_{Rényi}(I_Q, I_R, 2)$ is strictly positive, the corresponding similarity value satisfies

$$\begin{aligned} S_{Rényi}(I_Q, I_R, 2) &= 1 - \frac{J_{Rényi}(I_Q, I_R, 2)}{N_{Rényi}(I_Q, I_R, 2)} \\ &> 1. \end{aligned}$$

Thus, when $q > 1$ the term $J_{Rényi}$ can be negative, and the resulting similarity measure may lie outside the interval $[0, 1]$.

We now state the boundedness result for the range $q \in (0,1)$, where the Rényi entropy is concave.

Theorem 3. For $q \in (0,1)$, the Rényi entropy similarity measure $S_{Rényi}(I_Q, I_R, q)$ lies in $[0,1]$. Moreover, $S_{Rényi}(I_Q, I_R, q) = 1$ if and only if $I_Q = I_R$, and $S_{Rényi}(I_Q, I_R, q) = 0$ if and only if I_Q and I_R have disjoint support (i.e. $a_i b_i = 0$ for all i).

Proof. For $q \in (0,1)$, $H_{Rényi}(I, q)$ is concave, so Jensen's inequality gives

$$H_{Rényi}\left(\frac{I_Q + I_R}{2}, q\right) \geq \frac{1}{2} H_{Rényi}(I_Q, q) + \frac{1}{2} H_{Rényi}(I_R, q).$$

Thus $J_{Rényi}(I_Q, I_R, q) \geq 0$. Together with $N_{Rényi}(I_Q, I_R, q) > 0$, this shows that

$$S_{Rényi}(I_Q, I_R, q) = 1 - \frac{J_{Rényi}(I_Q, I_R, q)}{N_{Rényi}(I_Q, I_R, q)} \leq 1.$$

If $I_Q = I_R$, then $(I_Q + I_R)/2 = I_Q$ and hence $J_{Rényi}(I_Q, I_Q, q) = 0$, so $S_{Rényi}(I_Q, I_Q, q) = 1$.

To show $S_{Rényi} \geq 0$, it suffices to prove that $J_{Rényi}(I_Q, I_R, q) \leq N_{Rényi}(I_Q, I_R, q)$. This inequality is equivalent to

$$\ln\left(\sum_{i=1}^{n} \left(\frac{a_i + b_i}{2}\right)^q\right) \leq \ln\left(\sum_{i=1}^{n} \left(\frac{a_i}{2}\right)^q + \sum_{i=1}^{n} \left(\frac{b_i}{2}\right)^q\right).$$

Since the natural logarithm is increasing on $(0, \infty)$, it is enough to show that

$$\sum_{i=1}^{n} \left(\frac{a_i + b_i}{2}\right)^q \leq \sum_{i=1}^{n} \left(\frac{a_i}{2}\right)^q + \sum_{i=1}^{n} \left(\frac{b_i}{2}\right)^q.$$

For $q \in (0,1)$, the function x^q is concave on $[0, \infty)$, and one obtains for all $x, y \geq 0$,

$$(x + y)^q \leq x^q + y^q.$$

Applying this with $x = a_i$ and $y = b_i$ and then multiplying by 2^{-q} yields

$$\left(\frac{a_i + b_i}{2}\right)^q \leq \left(\frac{a_i}{2}\right)^q + \left(\frac{b_i}{2}\right)^q.$$

Summing over i gives the desired inequality, so $J_{Rényi} \leq N_{Rényi}$ and hence $S_{Rényi} \geq 0$.

Finally, we identify when $S_{Rényi} = 0$. By definition, $S_{Rényi}(I_Q, I_R, q) = 0$ if and only if $J_{Rényi}(I_Q, I_R, q) = N_{Rényi}(I_Q, I_R, q)$, which is equivalent to equality in

$$\sum_{i=1}^{n} \left(\frac{a_i + b_i}{2}\right)^q \leq \sum_{i=1}^{n} \left(\frac{a_i}{2}\right)^q + \sum_{i=1}^{n} \left(\frac{b_i}{2}\right)^q,$$

that is,

$$\sum_{i=1}^{n}\left(\frac{a_i+b_i}{2}\right)^q=\sum_{i=1}^{n}\left(\frac{a_i}{2}\right)^q+\sum_{i=1}^{n}\left(\frac{b_i}{2}\right)^q.$$

For $q\in(0,1)$, the function x^q is strictly concave on $[0,\infty)$, so for any $a_i,b_i\geq 0$ with $a_i>0$ and $b_i>0$,

$$\left(\frac{a_i+b_i}{2}\right)^q<\left(\frac{a_i}{2}\right)^q+\left(\frac{b_i}{2}\right)^q.$$

Thus, each index i with $a_i>0$ and $b_i>0$ contributes a strict inequality in the sum. Therefore, equality of the total sums can only hold if there is no such index, i.e. if for every i at least one of a_i or b_i is zero. Equivalently,

$$a_ib_i=0 \qquad \text{for all } i,$$

which means that I_Q and I_R have disjoint support. This completes the proof. □

References

[1] Hunter Dlugas, Xiang Zhang, and Seongho Kim. "Comparative analysis of continuous similarity measures for compound identification in mass spectrometry-based metabolomics". In: *Chemometrics and Intelligent Laboratory Systems* 263.105417 (Aug. 2025), p. 105417. DOI: 10.1016/j.chemolab.2025.105417.

[2] Yuanyue Li et al. "Spectral entropy outperforms MS/MS dot product similarity for small-molecule compound identification". In: *Nature Methods* 18.12 (2021), pp. 1524–1531. ISSN: 1548-7105. DOI: 10.1038/s41592-021-01331-z.

[3] J. Lin. "Divergence measures based on the Shannon entropy". In: *IEEE Transactions on Information Theory* 37.1 (1991), pp. 145–151. DOI: 10.1109/18.61115.

Table S1. Comparison of publicly available software packages for compound identification and annotation in metabolomics

Feature	Matchms	MetaboAnnotation	metID	ShinyMetID	PyCompound
Programming language	Python	R (Bioconductor ecosystem)	R	R/Shiny	Python
User interface	Programmatic (script/notebook) and Python package	Programmatic (script/notebook)	Programmatic (script/notebook)	Graphical user interface (GUI)	Programmatic (script/notebook), Python package, command-line interface (CLI), and Shiny GUI
Primary MS platform	MS/MS (LC-MS/MS and general MS/MS)	LC-MS (untargeted metabolomics)	LC-MS (metabolomics and exposomics)	GC-MS (EI-MS); applicable to LC-MS	Both high-resolution MS (HRMS; e.g., LC-MS/MS) and nominal-resolution MS (NRMS; e.g., GC-MS)
Main scope	Spectral preprocessing, similarity computation, and large-scale library matching	Modular MS1/MS2 annotation workflows	End-to-end automated LC-MS annotation	Interactive GC-MS spectral library matching	Unified spectral library matching with customizable preprocessing and systematic parameter tuning
MS1 (m/z) matching	Yes (precursor/parent mass filters)[a]	Yes (`matchValues`/`matchMz`; adduct-aware)	Yes (MSI level 3; public MS1 databases)	No (spectral matching only)	Yes (precursor-ion m/z filtering for HRMS only)
Retention time (RT) matching	Limited (metadata-based)	Yes (m/z + RT joint matching)	Yes (in-house database; same LC gradient required)	No	No
MS/MS / spectral matching	Yes (core functionality)	Yes (`matchSpectra`)	Yes (MSI levels 1–2; in-house and public MS2 databases)	Yes (library search)	Yes
Similarity measures	Multiple built-in: cosine (greedy/Hungarian), modified cosine, neutral-loss cosine, spectral (Shannon) entropy, fingerprint-based, metadata-based; extensible	Multiple built-in: normalized dot product (cosine-related), Euclidean, absolute-value distance, spectra angle; forward/reverse scores; spectral entropy via external packages; extensible	Single primary score: weighted dot product (forward + reverse + matched-fragment fraction; MS-DIAL-type algorithm)	Six measures: cosine, weighted cosine, Stein–Scott composite, DFT composite, DWT composite, partial/semi-partial correlation	Cosine, Shannon, Tsallis, and Rényi entropy; 15+ binary measures; user-defined mixture/composite measures
Spectral preprocessing	Extensive filter pipeline (peak filtering, normalization, metadata harmonization, precursor removal, etc.)	Via `Spectra` processing and peak-mapping strategies (`joinPeaks`, GNPS hybrid)	RT correction; intensity normalization for MS2 scoring	Weight-factor transformation (parameters a, b)	Filtering, weight-factor transformation, low-entropy transformation, centroiding, noise removal, peak matching
Customizable preprocessing workflows	Yes (composable filter pipelines; exportable as YAML)	Yes (modular parameter objects; custom `FUN`/`MAPFUN`)	Limited (fixed automated pipeline)	Limited (transformation weights; measure selection)	Yes (user-defined order of 2–6 preprocessing steps)
Systematic parameter tuning	Yes (tolerance, ppm, intensity weighting, entropy/cosine settings, etc.)	Yes (tolerance, ppm, RT window, score thresholds; stored in result objects)	Yes (ppm, RT tolerance, score weights/thresholds)	Yes (weight factors, rank, top-k hits)	Yes (exhaustive grid search and differential evolution optimization)
Workflow automation	User-built reproducible pipelines	User-built modular workflows; integrates with `xcms`	Fully automated annotation (`identify_metabolites`/`metIdentification`)	Interactive, step-by-step GUI	Package/CLI for batch processing; GUI for interactive use
In-house database construction	User-managed spectral import	Via `CompoundDb` and custom backends	Yes (`construct_database`; MSI level 1)	Custom library upload	Yes (`build_library_from_raw_data`)
Built-in/public reference libraries	None (user-supplied)	HMDB, MassBank, etc. (via ecosystem packages)	Multiple bundled public MS1/MS2 databases plus lab in-house database	NIST WebBook (21,166 spectra) and HMDB GC-MS (10,098 spectra)	GNPS and NIST WebBook (full libraries on Zenodo)
Supported input formats	mzML, mzXML, MGF, MSP, JSON	mzML, mzXML, netCDF, MGF, MSP, MassBank	MGF, MSP, mzXML	User-uploaded query/library files	MGF, mzML, MSP, CDF, JSON, TXT
Batch/large-scale processing	Yes (optimized for large libraries; sparse matrices)	Yes (native parallelization via `BiocParallel`)	Yes (multi-threaded)	Interactive (not designed for high-throughput batch)	Yes (CLI; multi-CPU differential evolution)
Extensibility	High (custom filters and similarity measures)	High (modular ecosystem; plug-in similarity functions)	Moderate (pipeline-oriented; single scoring framework)	Moderate (additional libraries/measures can be added)	High (mixture measures, custom weights, modular functions)
Integrated MSI annotation levels	No formal MSI workflow	No formal MSI workflow	Yes (MSI levels 1–3)	No formal MSI workflow	No formal MSI workflow

[a] Precursor/parent mass-based filtering rather than full MS1 feature annotation.
MetaboAnnotation: Rainer, J., et al. (2022). *Metabolites*, 12(2), 173.
metID: Shen, X., et al. (2022). *Bioinformatics*, 38(2), 568–569.
ShinyMetID: Oh, Y., et al. (2023). *Chemometrics and Intelligent Laboratory Systems*, 240, 104861.
GC-MS: gas chromatography–mass spectrometry; LC-MS: liquid chromatography–mass spectrometry; MS/MS: tandem mass spectrometry; MSI: Metabolomics Standards Initiative; GUI: graphical user interface; RT: retention time; HRMS: high-resolution mass spectrometry; NRMS: nominal-resolution mass spectrometry; CLI: command-line interface; DE: differential evolution; DFT: discrete Fourier transform; DWT: discrete wavelet transform.

Shiny Application Workflow

PyCompound is distributed as a Python package and command-line interface for scripted and batch analyses, and as an interactive Shiny web application for graphical exploration of spectral-library matching workflows. The publicly hosted Shiny application is available at `https://connect.posit.cloud/fy7392`, and a local version can be launched from the GitHub repository using `shiny run --launch-browser app.py`. Video tutorials demonstrating installation, data upload, analysis-mode selection, and result interpretation are available at `https://www.youtube.com/@PyCompound`. The Shiny application implements the same preprocessing procedures, similarity measures, and parameter-optimization strategies described in the main text and supports both NRMS (e.g., GC-MS) and HRMS (e.g., LC-MS/MS) workflows. Table S2 summarizes the four analysis modes, required inputs, primary outputs, and labeled-data requirements.

Plot and compare spectra

Users upload or select a query spectrum and a reference spectrum from a user-provided library. PyCompound displays the spectra before and after the selected preprocessing workflow, enabling visual assessment of peak alignment, intensity redistribution, and the effect of preprocessing order on spectral appearance. This mode is useful for inspecting preprocessing settings prior to library matching or parameter optimization. When compound identifiers are available, the interface provides direct links to PubChem entries for external chemical annotation and metadata.

Compound identification

Users upload query spectra and a reference spectral library, select the chromatography platform (NRMS or HRMS), choose one or more similarity measures, and specify preprocessing parameters and their execution order. PyCompound preprocesses each query spectrum, compares it against the reference library, and returns a ranked list of candidate matches with similarity scores. For each query spectrum, the top matched reference spectrum is displayed together with the query spectrum for visual inspection. PubChem links are provided when compound identifiers can be mapped to PubChem entries.

Parameter tuning by grid search

This mode requires labeled query spectra with known compound identities. Users specify discrete candidate values for one or more preprocessing or similarity parameters, and PyCompound evaluates all combinations of the selected parameter grid. For each parameter configuration, the complete identification workflow is executed and classification accuracy is computed as the proportion of query spectra correctly identified by the top-ranked library match.

Parameter tuning by differential evolution

This mode also requires labeled query spectra with known identities. Users select continuous parameters to optimize and specify lower and upper bounds for each parameter. PyCompound applies a differential evolution (DE) strategy to identify parameter values that maximize annotation accuracy without enumerating all possible combinations.

Example datasets for each analysis mode are provided in the `tests/data` directory of the PyCompound GitHub repository (`https://github.com/hdlugas/pycompound`). Full reference libraries are available from the associated Zenodo repository (`https://zenodo.org/records/12786324`).

Table S2. Analysis modes implemented in the PyCompound Shiny application

Analysis mode	Primary purpose	Required inputs	Primary outputs	Labeled query data required?
Plot and compare spectra	Visual inspection of query and reference spectra before/after preprocessing	Query spectrum; reference spectrum or library; preprocessing settings	Mirror-style spectrum plots; preprocessing summary; PubChem links (if available)	No
Compound identification	Ranked spectral-library matching and candidate annotation	Query spectra; reference library; platform (NRMS/HRMS); similarity measure(s); preprocessing settings	Ranked candidate matches; similarity scores; top-match spectrum plot; PubChem links (if available)	No
Parameter tuning (grid search)	Exhaustive evaluation of discrete parameter combinations	Labeled query spectra; reference library; parameter grid; similarity measure(s); preprocessing settings	Accuracy for each parameter combination; best parameter set; identification results under optimal settings	Yes
Parameter tuning (differential evolution)	Optimization of continuous parameters without exhaustive enumeration	Labeled query spectra; reference library; parameters to optimize; parameter bounds; similarity measure(s); preprocessing settings	Optimized parameter values; maximized accuracy; identification results under optimized settings	Yes

NRMS, nominal-resolution mass spectrometry; HRMS, high-resolution mass spectrometry; DE, differential evolution.

Table S3. Maximum Accuracies, 95% Confidence Intervals, and Optimal Parameters for ESI-based identification

Preprocessing Order	Configuration	Cosine	Shannon	Tsallis	Rényi (unbounded)	Rényi (bounded)
FWCNML	All	65.31% [63.41%,67.34%]<br>(0.70,0.00,3,1.30,0.55)	67.74% [65.97%,69.55%]<br>(0.70,0.00,3,0.00,1.00)	67.96% [66.15%,69.82%]<br>(0.70,0.00,3,0.00,1.00,1.10)	68.05% [66.20%,70.08%]<br>(0.70,0.00,3,0.00,1.00,1.10)	67.48% [65.58%,69.51%]<br>(0.70,0.00,3,0.00,1.00,0.95)
	No NR	65.31% [63.41%,67.34%]<br>(0.70,0.00,3,1.30,0.55)	67.74% [65.97%,69.55%]<br>(0.70,0.00,3,0.00,1.00)	67.96% [66.15%,69.82%]<br>(0.70,0.00,3,0.00,1.00,1.10)	68.05% [66.20%,70.08%]<br>(0.70,0.00,3,0.00,1.00,1.10)	67.48% [65.58%,69.51%]<br>(0.70,0.00,3,0.00,1.00,0.95)
	No WFT	64.47% [62.70%,66.42%]<br>(0.70,0.00,3,0.00,1.00)	67.74% [65.97%,69.55%]<br>(0.70,0.00,3,0.00,1.00)	67.96% [66.15%,69.82%]<br>(0.70,0.00,3,0.00,1.00,1.10)	68.05% [66.20%,70.08%]<br>(0.70,0.00,3,0.00,1.00,1.10)	67.48% [65.58%,69.51%]<br>(0.70,0.00,3,0.00,1.00,0.95)
	No LET	62.13% [60.14%,64.12%]<br>(0.70,0.00,0,1.30,0.55)	65.58% [63.68%,67.44%]<br>(0.70,0.01,0,1.30,0.55)	66.33% [64.52%,68.18%]<br>(0.90,0.01,0,0.00,1.00,0.50)	65.67% [63.63%,67.65%]<br>(0.70,0.01,0,1.30,0.55,0.90)	65.67% [63.63%,67.65%]<br>(0.70,0.01,0,1.30,0.55,0.90)
	No NR or LET	62.13% [60.14%,64.12%]<br>(0.70,0.00,0,1.30,0.55)	65.53% [63.59%,67.39%]<br>(0.70,0.00,0,1.30,0.55)	66.15% [64.34%,68.01%]<br>(0.70,0.00,0,0.00,1.00,0.50)	65.62% [63.63%,67.79%]<br>(0.70,0.00,0,1.30,0.55,1.05)	65.58% [63.59%,67.52%]<br>(0.70,0.00,0,1.30,0.55,0.90)
	No NR or WFT	64.47% [62.70%,66.42%]<br>(0.70,0.00,3,0.00,1.00)	67.74% [65.97%,69.55%]<br>(0.70,0.00,3,0.00,1.00)	67.96% [66.15%,69.82%]<br>(0.70,0.00,3,0.00,1.00,1.10)	68.05% [66.20%,70.08%]<br>(0.70,0.00,3,0.00,1.00,1.10)	67.48% [65.58%,69.51%]<br>(0.70,0.00,3,0.00,1.00,0.95)
	No LET or WFT	60.54% [58.55%,62.40%]<br>(0.70,0.01,0,0.00,1.00)	64.83% [62.97%,66.68%]<br>(0.70,0.00,0,0.00,1.00)	66.33% [64.52%,68.18%]<br>(0.90,0.01,0,0.00,1.00,0.50)	65.31% [63.37%,67.35%]<br>(0.70,0.00,0,0.00,1.00,0.50)	65.31% [63.37%,67.35%]<br>(0.70,0.00,0,0.00,1.00,0.50)
	No NR, LET, or WFT	60.49% [58.55%,62.31%]<br>(0.70,0.00,0,0.00,1.00)	64.83% [62.97%,66.68%]<br>(0.70,0.00,0,0.00,1.00)	66.15% [64.34%,68.01%]<br>(0.70,0.00,0,0.00,1.00,0.50)	65.31% [63.37%,67.35%]<br>(0.70,0.00,0,0.00,1.00,0.50)	65.31% [63.37%,67.35%]<br>(0.70,0.00,0,0.00,1.00,0.50)
FCNWML	All	68.98% [67.21%,70.93%]<br>(0.70,0.00,3,1.30,0.55)	67.87% [65.93%,69.73%]<br>(0.50,0.00,3,1.30,0.55)	68.94% [67.03%,70.79%]<br>(0.50,0.01,3,1.10,0.53,1.50)	68.54% [66.64%,70.53%]<br>(0.50,0.00,3,1.30,0.55,1.15)	67.83% [65.93%,69.86%]<br>(0.70,0.01,0,1.30,0.55,0.95)
	No NR	68.98% [67.21%,70.93%]<br>(0.70,0.00,3,1.30,0.55)	67.87% [65.93%,69.73%]<br>(0.50,0.00,3,1.30,0.55)	68.89% [66.95%,70.79%]<br>(0.50,0.00,3,1.10,0.53,1.50)	68.54% [66.64%,70.53%]<br>(0.50,0.00,3,1.30,0.55,1.15)	67.61% [65.71%,69.55%]<br>(0.70,0.00,0,1.10,0.53,0.95)
	No WFT	64.47% [62.70%,66.42%]<br>(0.70,0.00,3,0.00,1.00)	67.74% [65.97%,69.55%]<br>(0.70,0.00,3,0.00,1.00)	67.96% [66.15%,69.82%]<br>(0.70,0.00,3,0.00,1.00,1.10)	68.05% [66.20%,70.08%]<br>(0.70,0.00,3,0.00,1.00,1.10)	67.48% [65.58%,69.51%]<br>(0.70,0.00,3,0.00,1.00,0.95)
	No LET	66.24% [64.30%,68.10%]<br>(0.70,0.00,0,1.10,0.51)	67.87% [65.97%,69.86%]<br>(0.70,0.01,0,1.10,0.51)	67.92% [66.02%,69.91%]<br>(0.70,0.01,0,1.10,0.53,0.95)	68.10% [66.20%,70.13%]<br>(0.50,0.01,0,1.10,0.53,1.15)	67.83% [65.93%,69.86%]<br>(0.70,0.01,0,1.30,0.55,0.95)
	No NR or LET	66.24% [64.30%,68.10%]<br>(0.70,0.00,0,1.10,0.51)	67.79% [65.84%,69.77%]<br>(0.70,0.00,0,1.10,0.51)	67.83% [65.93%,69.82%]<br>(0.70,0.00,0,1.10,0.53,0.95)	67.96% [66.02%,69.95%]<br>(0.70,0.00,0,1.10,0.51,1.05)	67.61% [65.71%,69.55%]<br>(0.70,0.00,0,1.10,0.53,0.95)
	No NR or WFT	64.47% [62.70%,66.42%]<br>(0.70,0.00,3,0.00,1.00)	67.74% [65.97%,69.55%]<br>(0.70,0.00,3,0.00,1.00)	67.96% [66.15%,69.82%]<br>(0.70,0.00,3,0.00,1.00,1.10)	68.05% [66.20%,70.08%]<br>(0.70,0.00,3,0.00,1.00,1.10)	67.48% [65.58%,69.51%]<br>(0.70,0.00,3,0.00,1.00,0.95)
	No LET or WFT	60.54% [58.55%,62.40%]<br>(0.70,0.01,0,0.00,1.00)	64.83% [62.97%,66.68%]<br>(0.70,0.00,0,0.00,1.00)	66.33% [64.52%,68.18%]<br>(0.90,0.01,0,0.00,1.00,0.50)	65.31% [63.37%,67.35%]<br>(0.70,0.00,0,0.00,1.00,0.50)	65.31% [63.37%,67.35%]<br>(0.70,0.00,0,0.00,1.00,0.50)
	No NR, LET, or WFT	60.49% [58.55%,62.31%]<br>(0.70,0.00,0,0.00,1.00)	64.83% [62.97%,66.68%]<br>(0.70,0.00,0,0.00,1.00)	66.15% [64.34%,68.01%]<br>(0.70,0.00,0,0.00,1.00,0.50)	65.31% [63.37%,67.35%]<br>(0.70,0.00,0,0.00,1.00,0.50)	65.31% [63.37%,67.35%]<br>(0.70,0.00,0,0.00,1.00,0.50)
FCNMWL	All	69.07% [67.26%,71.01%]<br>(0.70,0.00,3,1.30,0.55)	67.92% [66.02%,69.95%]<br>(0.70,0.01,0,1.10,0.51)	68.94% [67.03%,70.84%]<br>(0.50,0.00,3,1.10,0.53,1.50)	68.63% [66.72%,70.66%]<br>(0.50,0.00,3,1.30,0.55,1.15)	67.87% [65.97%,69.86%]<br>(0.70,0.01,0,1.30,0.55,0.95)
	No NR	69.07% [67.26%,71.01%]<br>(0.70,0.00,3,1.30,0.55)	67.87% [65.93%,69.73%]<br>(0.50,0.00,3,1.30,0.55)	68.94% [67.03%,70.84%]<br>(0.50,0.00,3,1.10,0.53,1.50)	68.63% [66.72%,70.66%]<br>(0.50,0.00,3,1.30,0.55,1.15)	67.70% [65.80%,69.69%]<br>(0.70,0.00,0,1.10,0.51,0.95)
	No WFT	64.47% [62.70%,66.42%]<br>(0.70,0.00,3,0.00,1.00)	67.74% [65.97%,69.55%]<br>(0.70,0.00,3,0.00,1.00)	67.96% [66.15%,69.82%]<br>(0.70,0.00,3,0.00,1.00,1.10)	68.05% [66.20%,70.08%]<br>(0.70,0.00,3,0.00,1.00,1.10)	67.48% [65.58%,69.51%]<br>(0.70,0.00,3,0.00,1.00,0.95)
	No LET	66.33% [64.34%,68.18%]<br>(0.70,0.00,0,1.10,0.51)	67.92% [66.02%,69.95%]<br>(0.70,0.01,0,1.10,0.51)	67.96% [66.11%,69.95%]<br>(0.70,0.01,0,1.10,0.51,1.05)	68.10% [66.20%,70.13%]<br>(0.50,0.01,0,1.10,0.53,1.15)	67.87% [65.97%,69.86%]<br>(0.70,0.01,0,1.30,0.55,0.95)
	No NR or LET	66.33% [64.34%,68.18%]<br>(0.70,0.00,0,1.10,0.51)	67.83% [65.89%,69.82%]<br>(0.70,0.00,0,1.10,0.51)	67.87% [65.97%,69.86%]<br>(0.70,0.00,0,1.10,0.53,0.95)	68.01% [66.06%,70.00%]<br>(0.70,0.00,0,1.10,0.51,1.05)	67.70% [65.80%,69.69%]<br>(0.70,0.00,0,1.10,0.51,0.95)
	No NR or WFT	64.47% [62.70%,66.42%]<br>(0.70,0.00,3,0.00,1.00)	67.74% [65.97%,69.55%]<br>(0.70,0.00,3,0.00,1.00)	67.96% [66.15%,69.82%]<br>(0.70,0.00,3,0.00,1.00,1.10)	68.05% [66.20%,70.08%]<br>(0.70,0.00,3,0.00,1.00,1.10)	67.48% [65.58%,69.51%]<br>(0.70,0.00,3,0.00,1.00,0.95)
	No LET or WFT	60.54% [58.55%,62.40%]<br>(0.70,0.01,0,0.00,1.00)	64.83% [62.97%,66.68%]<br>(0.70,0.00,0,0.00,1.00)	66.33% [64.52%,68.18%]<br>(0.90,0.01,0,0.00,1.00,0.50)	65.31% [63.37%,67.35%]<br>(0.70,0.00,0,0.00,1.00,0.50)	65.31% [63.37%,67.35%]<br>(0.70,0.00,0,0.00,1.00,0.50)
	No NR, LET, or WFT	60.49% [58.55%,62.31%]<br>(0.70,0.00,0,0.00,1.00)	64.83% [62.97%,66.68%]<br>(0.70,0.00,0,0.00,1.00)	66.15% [64.34%,68.01%]<br>(0.70,0.00,0,0.00,1.00,0.50)	65.31% [63.37%,67.35%]<br>(0.70,0.00,0,0.00,1.00,0.50)	65.31% [63.37%,67.35%]<br>(0.70,0.00,0,0.00,1.00,0.50)

FWCNML: filtering → weight factor transformation → centroid → noise removal → match → low-entropy transformation.
FCNWML: filtering → centroid → noise removal → weight factor transformation → match → low-entropy transformation.
FCNMWL: filtering → centroid → noise removal → match → weight factor transformation → low-entropy transformation.
NR: noise removal.
WFT: weight factor transformation.
LET: low-entropy transformation.
The number outside of square brackets and parentheses represent accuracy.
The numbers in square brackets represent 95% confidence interval.
The numbers within the parentheses represent the optimal parameters.
Parameter order: (window-size, NR, LET, WF-m/z, WF-intensity, entropy dimension).
Entropy dimension only applies to Tsallis and Rényi Entropy Similarity Measures.

Table S4. Maximum Accuracies, 95% Confidence Intervals, and Optimal Parameters for EI-based identification

Preprocessing Order	Configuration	Cosine	Shannon	Tsallis	Rényi (unbounded)	Rényi (bounded)
WL	All	84.28% [83.75%,84.73%]<br>(3,1.30,0.55)	80.69% [80.17%,81.25%]<br>(3,0.00,1.00)	81.68% [81.15%,82.19%]<br>(3,1.30,0.55,1.50)	81.09% [80.56%,81.63%]<br>(3,1.30,0.55,1.50)	80.30% [79.78%,80.83%]<br>(3,0.00,1.00,0.95)
	No WFT	75.94% [75.36%,76.56%]<br>(3,0.00,1.00)	80.69% [80.17%,81.25%]<br>(3,0.00,1.00)	80.84% [80.33%,81.38%]<br>(3,0.00,1.00,1.10)	80.97% [80.44%,81.50%]<br>(3,0.00,1.00,1.10)	80.30% [79.78%,80.83%]<br>(3,0.00,1.00,0.95)
	No LET	84.19% [83.65%,84.64%]<br>(0,1.30,0.55)	80.13% [79.64%,80.68%]<br>(0,0.00,1.00)	81.46% [80.91%,81.95%]<br>(0,1.30,0.55,1.50)	81.00% [80.45%,81.53%]<br>(0,1.30,0.55,1.25)	79.95% [79.43%,80.49%]<br>(0,0.00,1.00,0.95)
	No WFT and no LET	72.74% [72.12%,73.34%]<br>(0,0.00,1.00)	80.13% [79.64%,80.68%]<br>(0,0.00,1.00)	80.13% [79.61%,80.67%]<br>(0,0.00,1.00,0.95)	80.28% [79.76%,80.82%]<br>(0,0.00,1.00,1.10)	79.95% [79.43%,80.49%]<br>(0,0.00,1.00,0.95)

WL: weight factor transformation $\rightarrow$ low-entropy transformation.
WFT: weight-factor transformation.
LET: low-entropy transformation.
The number outside the square brackets and parentheses represents the annotation accuracy.
The numbers in square brackets represent the 95% confidence interval.
The numbers in parentheses represent the optimal parameter settings.
Parameter order: (LET, WF-m/z, WF-intensity, entropy dimension).
The entropy dimension applies only to the Tsallis and Rényi entropy similarity measures.
For the EI-based GC-MS dataset, centroiding, noise removal, and peak matching were not applied because the reference library spectra were already centroided and denoised, and fragment-ion m/z values are represented as nominal (integer) masses. Consequently, the preprocessing workflow consisted only of weight-factor transformation (W) followed by low-entropy transformation (L), i.e., WL.

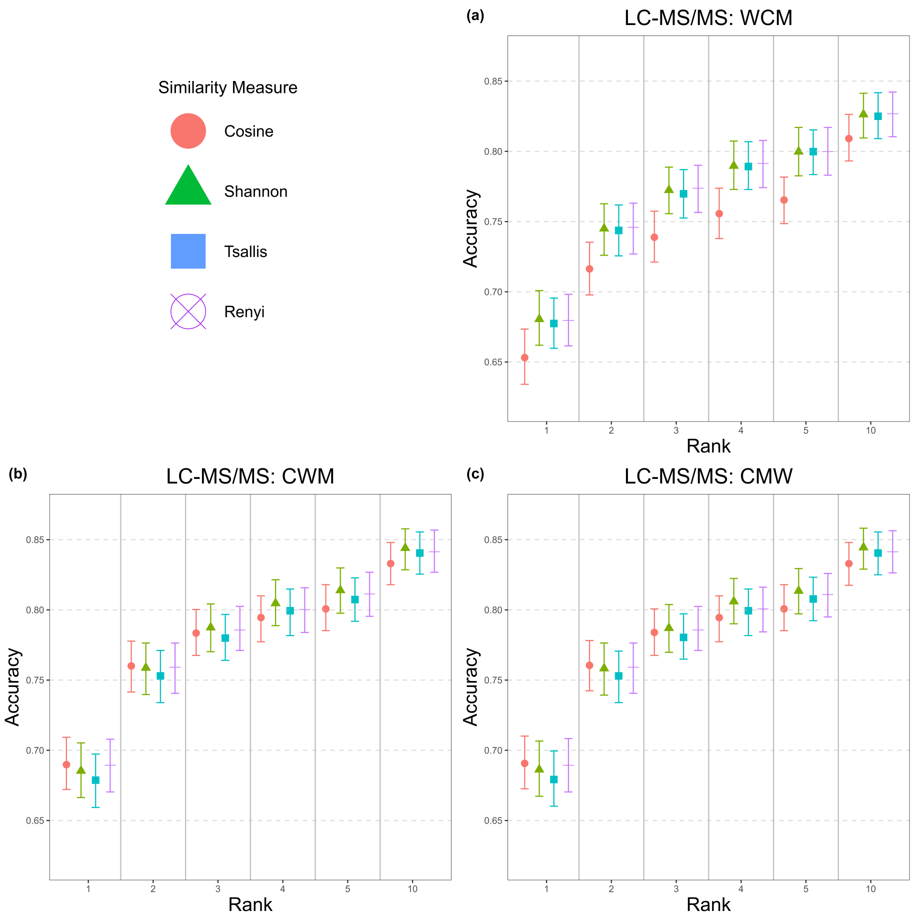

Figure S1: Top-n annotation accuracy for the GNPS LC-MS/MS dataset under the optimized preprocessing workflows and parameter settings. A query spectrum was considered correctly annotated at rank n if its correct compound appeared among the n reference-library compounds with the highest similarity scores. The preprocessing workflows were **(a)** FWCNML, **(b)** FCNWML, and **(c)** FCNMWL. F, filtering; W, weight-factor transformation; C, centroiding; N, noise removal; M, peak matching; and L, low-entropy transformation. The Rényi results shown in this figure correspond to the original unrestricted analysis, in which the entropy dimension α was optimized without restriction to $0 < \alpha < 1$. Results from the bounded Rényi analysis, in which α was restricted to $0 < \alpha < 1$, are provided in Figure 1D, Table S3, and Supporting Information 2.

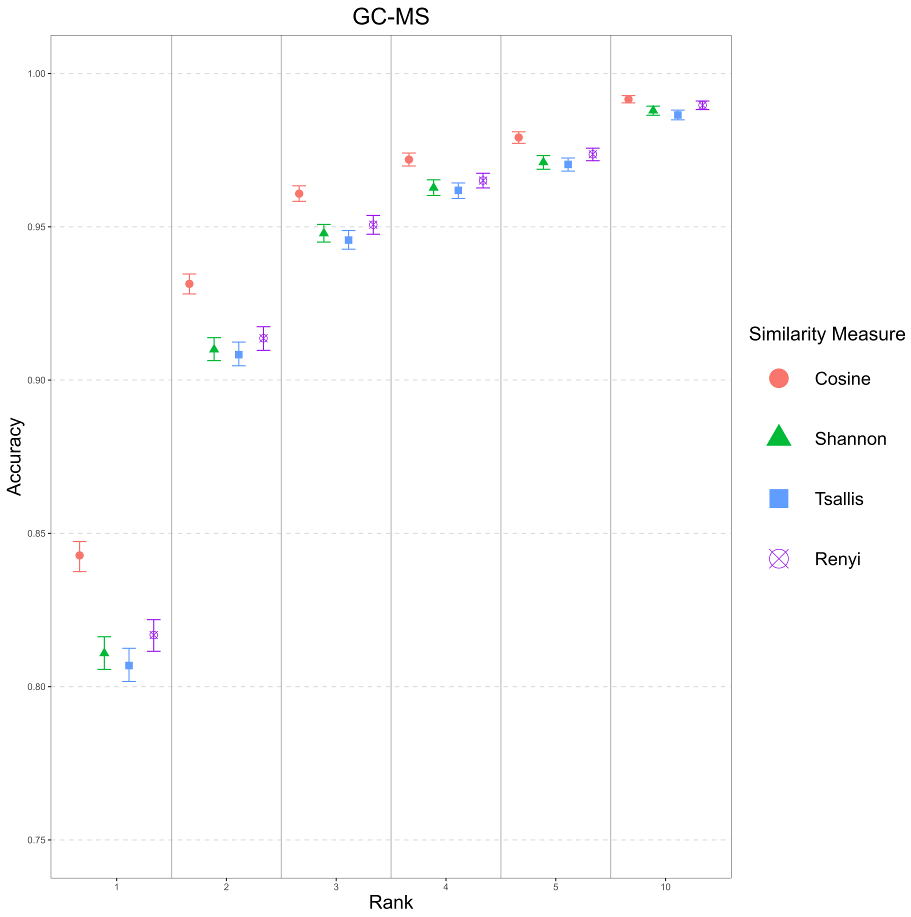

Figure S2: Top-n annotation accuracy for the WebNIST GC-MS dataset under the optimized preprocessing workflow and parameter settings. A query spectrum was considered correctly annotated at rank n if its correct compound appeared among the n reference-library compounds with the highest similarity scores. The Rényi results shown in this figure correspond to the original unrestricted analysis, in which the entropy dimension α was optimized without restriction to $0 < \alpha < 1$. Results from the bounded Rényi analysis, in which α was restricted to $0 < \alpha < 1$, are provided in Figure 1D, Table S4, and Supporting Information 2.
